# Ethnic minority and migrant underrepresentation in Covid-19 research: Causes and solutions

**DOI:** 10.1016/j.eclinm.2021.100903

**Published:** 2021-05-29

**Authors:** Melanie Etti, Hazel Fofie, Mohammad Razai, Alison F. Crawshaw, Sally Hargreaves, Lucy P. Goldsmith

**Affiliations:** a St George's University of London, London, UK; b Primary Care, St George's University of London, UK; c Migrant Health and Participatory Research, St George's University of London, London, UK; d Global Health, St George's University of London, London, UK; e Global Health, St George's University of London, London, UK

The Covid-19 pandemic has highlighted the longstanding under representation of ethnic minorities in clinical research – including migrant populations [1]. Ethnic minorities in the UK are at significantly greater risk of severe disease and death from Covid-19 than their White counterparts [2]. Similarly, migrants in high-income countries with Covid-19 have been identified as being at potentially greater risk of hospitalisation and death than non-migrants [3]. Despite this, a recent National Institute for Health Research (NIHR) report found ethnic minorities constitute only 9·26% of participants in UK Covid-19 studies, below their representation in the general population (13·80%) [4].

The NIHR has stated that Covid-19 research needs more ethnic minority participants to ensure the research is representative of the UK population. The inclusion of these groups in Covid-19 vaccine trials is particularly pertinent, with vaccine uptake being lower among ethnic minority groups than White people in the UK despite their increased risk [5]. Some migrant group's concerns around the vaccine are due to their lack of inclusion in the clinical trials, highlighting an urgent need to understand and address the reasons underlying these disparities in Covid-19 research participation [6].

Reasons for the underrepresentation of ethnic minority groups in Covid-19 research are poorly elucidated but are likely due to a combination of personal and structural factors. Socio-political factors may include social deprivation limiting access to health services, and subsequently, participation in – and awareness of – health research. Participant-related factors may include language and cultural barriers, and mistrust towards researchers and research institutions. Fear, mistrust and access barriers are the effects of powerful upstream factors including structural racism, marginalisation and ethnic exclusion. Previous studies have noted that a good researcher-participant relationship is a key facilitator of their participation [7]. Lack of ethnic minority participation has also been linked to racial discrimination and previous negative healthcare experiences [5]. A qualitative study in the UK seeking the views of ethnic minorities towards participation in Covid-19 vaccine trials identified a range of barriers including concerns around attending hospitals, lack of support if problems arose and language barriers between themselves and research staff, despite agreement that this research was necessary [8].

Factors relating to research design and approach taken by researchers, including the omission of ethnicity from covariate data and non-inclusive recruitment strategies, are also likely to impact negatively on recruitment from these communities. As of 27th March 2021, only 1·43% of US-based studies investigating Covid-19 registered on Clinicaltrials.gov were collecting data about ethnicity, highlighting how this problem continues to go overlooked. Another review examining ethnic minority research participation found that the number of individuals from minority groups actually invited by researchers to participate in research was disproportionately small compared to their representation in the wider population [9]. Strategies to increase ethnic minority representation in medical research must seek to tackle the root causes of underrepresentation including systemic racism, racial discrimination and access barriers.

In-depth cross-sectoral qualitative research is urgently needed to better understand the barriers hindering the involvement of people from ethnic minority communities in clinical research and develop robust solutions. Initiatives such as the INCLUDE project, which aims to promote the inclusion of underserved communities in clinical trials, are forming the basis for this much needed work and may provide fresh insight and useful action points [10]. Researchers and policy makers should be educated and supported to prioritise equitable access to research participation for ethnic minorities and migrants, particularly for conditions that disproportionately affect them. Strategies for overcoming participation disparities should not be ad hoc, but rather, part of a policy framework underpinned by Principle 13 of the Declaration of Helsinki. Practical solutions for increasing recruitment could include translating participant information into languages other than English, using diverse recruitment strategies to ensure study information and invitations reach potential participants among ethnic minority and migrant communities, and developing community engagement programmes to dispel rumours and misinformation which may erode trust in these studies [8]. Addressing factors connected to economic disadvantage (which may disproportionately affect certain ethnic minority groups) will also be needed, including reimbursement for childcare costs and travel expenses [9]. Being clear about the potential benefits of participation will be important, alongside consideration of cultural and religious schedules [8].

The NHS Race & Health Observatory, which has responded to the specific health and wellbeing of ethnic groups in the UK, should now also focus on the equitable inclusion of these groups in Covid-19 clinical research and trials. Ultimately, we should ensure meaningful participation is placed at the heart of public health interventions research, with beneficiaries of the research included in every stage of the research pathway [6].

## Declaration of Competing Interest

The Authors have nothing to disclose. The views expressed are those of the author(s) and not necessarily those of the NIHR or the Department of Health and Social Care.
